# Preserved Fat-Free Mass after Gastric Bypass and Duodenal Switch

**DOI:** 10.1007/s11695-016-2476-6

**Published:** 2016-11-24

**Authors:** Martin Skogar, Ulf Holmbäck, Jakob Hedberg, Ulf Risérus, Magnus Sundbom

**Affiliations:** 1 Department of Surgical Sciences, Uppsala University, SE-751 85 Uppsala, Sweden; 2 Department of Public Health and Caring Sciences, Clinical Nutrition and Metabolism, Uppsala University, SE-751 85 Uppsala, Sweden

**Keywords:** Body composition, Fat-free mass, Resting metabolic rate, Bariatric surgery, Obesity, Roux-en-Y gastric bypass, Duodenal switch, Air-displacement plethysmography, Bod Pod, Indirect calorimetry assessment

## Abstract

**Background:**

Concerns for the possibility of an excessive loss of fat-free mass (FFM) and resting metabolic rate (RMR) after bariatric surgery, such as Roux-en-Y gastric bypass (RYGB) and duodenal switch (BPD/DS), have been raised.

**Objectives:**

This study aims to examine body composition and RMR in patients after RYGB and BPD/DS and in non-operated controls.

**Methods:**

Body composition and RMR were studied with Bod Pod and indirect calorimetry in weight-stable RYGB (*n* = 15) and BPD/DS patients (*n* = 12) and compared with non-operated controls (*n* = 17). All patients were 30–55 years old and weight stable with BMI 28–35 kg/m^2^.

**Results:**

FFM% was 58% (RYGB), 61% (BPD/DS), and 58% (controls). Body composition did not differ after RYGB and BPD/DS compared to controls, despite 27 and 40% total body weight loss, respectively. No difference in RMR or RMR/FFM was observed (1539, 1617, and 1490 kcal/24 h; and 28.9, 28.4, and 28.8 kcal/24 h/kg).

**Conclusion:**

Weight-stable patients with BMI 28–35 kg/m^2^ after RYGB and BPD/DS have a body composition and RMR similar to that of non-operated individuals within the same BMI interval.

## Introduction

Long-term results after bariatric surgery include sustained weight loss, high remission rates in diabetes, improved cardiovascular risk profile (reduced hypertension and dyslipidemia), and improved quality of life [1–4]. Roux-en-Y gastric bypass (RYGB) is a common surgical procedure for the treatment of severe obesity (body mass index (BMI) > 35 kg/m^2^) worldwide. In Sweden, RYGB represents 82% of all bariatric procedures in 2015 [5], while duodenal switch (BPD/DS) is mostly offered in patients with super obesity (BMI > 50 kg/m^2^).

Loss of fat mass (FM) in particular, while preserving fat-free mass (FFM), is desirable when treating patients with severe obesity. Non-adipose tissues, for example muscles, are not only responsible for maintaining the body’s functional capacity but their maintenance also requires energy in the resting state, i.e., the resting metabolic rate (RMR) [6]. As RMR constitutes approximately 70% of the total daily energy expenditure [7], the long-term effect on weight loss after bariatric surgery can be affected by the amount of remaining FFM. Concerns for the possibility of malnutrition and an excessive loss of FFM after RYGB and especially BPD/DS have been raised and the safety in regard to nutrition and body composition are considered potentially problematic [8].

The aim of this study was to examine body composition and RMR in weight-stable patients after RYGB and BPD/DS and to compare these two groups to non-operated controls.

## Subjects and Methods

### Subjects

Weight-stable RYGB and BPD/DS patients between 30 and 55 years, operated on at least 2 years previously, with a BMI of 28–35 kg/m^2^ at their last checkup, were identified from a local database (Department of Surgery, Uppsala University Hospital, Sweden) and contacted retrospectively by mail. Eighteen RYGB and 15 BPD/DS patients accepted to participate in the study; however, 6 patients (3 RYGB and 3 BPD/DS) had to be excluded as they had a BMI >35 kg/m^2^ at measurement. Seventeen patients within the same BMI interval (28–35 kg/m^2^) and age from a previous study [9] were used as controls. The final study groups thus consisted of 15 RYGB, 12 BPD/DS, and 17 controls.

### Measurements

All patients underwent body composition analysis by air-displacement plethysmography (Bod Pod; Body Composition System, Cosmed, Rome, Italy) after an overnight fast (water allowed from midnight). In a Bod Pod, the volume of an individual is measured indirectly by measuring the volume of air the body displaces inside an enclosed chamber (plethysmograph). Body weight and body volume were measured in the Bod Pod and body density was calculated. Body density was then used for estimating body fat based on a two-compartment model, which divides the body into FM and FFM [10]. FM, FFM, percent fat mass (FM%), and percent fat-free mass (FFM%) were obtained from the Bod Pod measurements. In addition, the Bod Pod gives an estimation of RMR (kcal/24 h) based on the relationship between FM and FFM, using the Nelson Prediction Equation [RMR (kcal/24 h) = 25.80 × fat-free mass (kg) + 4.04 × fat mass (kg)]. All measurements were performed at the same temperature. Subjects were dressed in underwear and used a swim cap to tuck in their hair.

In the two bariatric groups (RYGB and BPD/DS), RMR was also measured with indirect calorimetry (Vmax Encore 29N; CareFusion, San Diego, USA) according to standard clinical practice [7]. As RMR was obtained with two methods, Bod Pod and indirect calorimetry (often considered “gold standard”), a comparison between the two methods was possible.

### Statistics

Values are presented as mean ± standard deviation (SD) unless otherwise stated. A *p* value of <0.05 was considered statistically significant. For comparison of parametric data, analysis of variance (ANOVA) was used. Tukey’s method was used for simultaneous post hoc comparison of means when comparing the three groups. A chi-square test was used to evaluate for possible differences in gender distribution. When comparing two groups, an unpaired *t* test was used. GraphPad Prism version 6.0f (GraphPad Software, La Jolla, California, USA) was used for the statistical analysis.

### Ethics

The Regional Ethical Board at Uppsala University approved the study, and all subjects signed an informed consent form (Dnr 2008/154).

## Results

### General

Preoperative BMI was higher in the BPD/DS group compared with the RYGB group (as expected based on selection of patients for BPD/DS, BMI >50 kg/m^2^). Total body weight loss was 27% for RYGB and 40% for BPD/DS. The measurements were performed on average 4.1 (range 2.9–8.3) and 6.4 (range 2.8–9.5) years postoperatively in RYGB and BPD/DS, respectively. There were no significant differences between the three groups concerning gender distribution, age, height, weight, or present BMI (Table 1).

**Table 1.** Descriptive statistics of subjects (mean ± SD)

	RYGB	BPD/DS	Controls	*p* value	*p* value	*p* value
	(*n* = 15)	(*n* = 12)	(*n* = 17)	RYGB vs. controls	BPD/DS vs. controls	RYGB vs. BPD/DS
Gender, male/female	3:12	5:7	3:14	0.865	0.154	0.221
Age (years)	45.9 ± 5.8	44.5 ± 7.3	45.7 ± 8.4	0.996	0.899	0.868
Height (m)	1.69 ± 0.10	1.71 ± 0.10	1.70 ± 0.08	0.989	0.860	0.799
Weight (kg)	93.8 ± 12.9	91.8 ± 12.8	89.4 ± 10.9	0.561	0.861	0.901
Present BMI (kg/m^2^)	32.7 ± 1.7	31.2 ± 2.1	31.0 ± 2.5	0.091	0.988	0.173
Preoperative BMI (kg/m^2^)	45.1 ± 3.4	52.2 ± 4.3	N/A	N/A	N/A	<0.001

*RYGB* Roux-en-Y gastric bypass, *BPD/DS* duodenal switch, *BMI* body mass index

### Body Composition

**Fig. 1.**
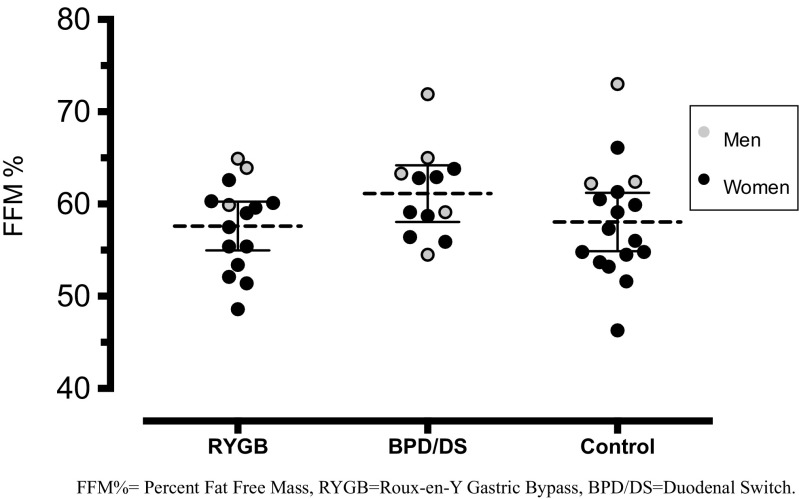
Groups are presented as mean with 95% CI of percent fat-free mass (FFM%)

There were no significant differences in FFM, FM, FFM/FM ratio, FFM%, or FM% between the three groups (Table 2). Both RYGB and BPD/DS had a similar FFM% as controls (Fig. 1). When comparing men and women separately, we found no difference in body composition between RYGB, BPD/DS, and controls, although the study was not powered for this.

**Table 2.** Body composition and resting metabolic rate (RMR): data from Bod Pod and indirect calorimetry (mean ± SD)

	RYGB	BPD/DS	Controls	*p* value	*p* value	*p* value
	(*n* = 15)	(*n* = 12)	(*n* = 17)	RYGB vs. controls	BPD/DS vs. controls	RYGB vs. BPD/DS
Bod Pod						
Weight (kg)	93.8 ± 12.9	91.8 ± 12.8	89.4 ± 10.9	0.561	0.861	0.901
FFM (kg)	54.2 ± 10.2	55.9 ± 7.5	51.9 ± 8.6	0.734	0.457	0.879
FM (kg)	39.6 ± 5.7	35.8 ± 7.7	37.5 ± 7.1	0.678	0.796	0.350
FFM/FM	1.39 ± 0.27	1.61 ± 0.37	1.44 ± 0.42	0.912	0.421	0.254
FFM %	57.6 ± 4.8	61.1 ± 4.8	58.0 ± 6.2	0.972	0.293	0.222
FM %	42.4 ± 4.8	38.9 ± 4.8	42.0 ± 6.2	0.972	0.293	0.222
RMR (kcal/24 h)	1539 ± 265 (*n* = 14)	1617 ± 194 (*n* = 11)	1490 ± 223 (*n* = 17)	0.828	0.339	0.680
RMR/FFM (kcal/24 h/kg)	28.9 ± 0.58	28.4 ± 0.56	28.8 ± 0.74	0.905	0.270	0.155
Indirect calorimetry						
RMR (kcal/24 h)	1670 ± 293	1689 ± 241	N/A	N/A	N/A	0.857
RMR/FFM (kcal/24 h/kg)	31.0 ± 3.0	30.3 ± 2.0	N/A	N/A	N/A	0.448

*RYGB* Roux-en-Y gastric bypass, *BPD/DS* duodenal switch, *FFM* fat-free mass, *FM* fat mass, *FFM %* percent fat-free mass, *FM %* percent fat mass

### Resting Metabolic Rate

No significant differences in RMR or RMR/FFM were found between the three groups as they were in line with controls for both RYGB and BPD/DS (Table 2).

When comparing RMR data from Bod Pod with indirect calorimetry in the bariatric patients (RYGB + BPD/DS), there was a weak tendency towards underestimation of RMR with Bod Pod (1569 ± 228 vs. 1674 ± 258 kcal/24 h, *p* = 0.12). Bod Pod calculations were found to underestimate RMR/FFM (28.7 ± 0.6 vs. 30.7 ± 2.5 kcal/24 h/kg, *p* < 0.001).

## Discussion

Among bariatric surgeons, concerns have been raised that BPD/DS yields a catabolic state, with significant nutritional risks, as the greater weight loss may be accompanied by greater loss of FFM. In this study, we wanted to examine weight-stable patients several years after surgery to see the long-term effects of bariatric surgery on body composition. Despite the massive postoperative weight loss after RYGB and BPD/DS (27 and 40% of total body weight loss, respectively), body composition did not differ from a non-operated control group with the same BMI (28–35 kg/m^2^) in this study. In addition, the present results are consistent with a study by Tacchino et al. [11], who found that 101 female BPD/DS patients had a body composition close to physiological values 2 years after surgery, when comparing with 53 weight-matched non-operated female controls. In a systematic review by Chaston et al. [8], RYGB and BPD/DS were found to cause greater percentage of FFM loss compared with restrictive bariatric surgery (laparoscopic adjustable gastric banding). However, the included studies in this review are difficult to compare due to several reasons, e.g., initial BMI and gender proportions varied, restrictive bariatric surgery resulted in less total body weight loss, and follow-up time was very diverse. In addition, statistical comparisons were made without weighting by the number of patients (*n*) included in each study. The authors state that future additional studies are needed to show the differences with statistical certainty. Benedetti et al. [12] found significantly higher FFM and RMR in 14 BPD/DS patients, 30 months postoperatively, compared with 14 matched controls (matched for age, gender, and height), possibly because of higher body weight in the BPD/DS group.

There is a significant reduction in FM, FFM, and RMR during weight loss [13–17]. A widely cited rule (“Quarter FFM Rule”), guiding expected loss of FFM during weight loss, states that the expected loss of FM will be approximately three fourths of the total weight loss and the remaining one fourth will be loss of FFM [18]. However, this is at best an approximation since the proportion of FFM loss varies over time during weight loss and depends on several factors such as degree of baseline adiposity, gender, level of physical activity, amount of energy intake, and diet composition [18–21]. After a 15% reduction in body weight, Kulovitz et al. [19] described a 3:1 ratio between FM and FFM loss both in an integrated medical therapy group and post-bariatric surgery. In contrast, Wadstrom et al. [20] reported a 1:1 ratio in FM/FFM loss during the rapid weight loss in the first 3 months after surgery (gastric banding and vertical banded gastroplasty), which was followed by continuous decrease in FM, while FFM was not reduced further.

As RMR accounts for the largest proportion of an individual’s total daily energy expenditure, it has been suggested to be an important predictor of weight maintenance after weight loss [7, 22]. Whether bariatric surgery also promotes weight loss through effects on RMR is uncertain. A recent study by Schmidt et al. [23] found a decrease in RMR after RYGB, while another study concluded that the increased postprandial RMR could be one mechanism that contributes to weight loss after RYGB [24]. This study suggests that RYGB and BPD/DS result in a RMR and RMR/FFM similar to that of non-operated controls with the same BMI (28–35 kg/m^2^). The lack of differences in body compositions is corroborated with the lack of differences in RMR, in our opinion.

In the present study, Bod Pod underestimated RMR/FFM compared to indirect calorimetry by roughly 7%. A weak tendency towards a difference in whole-body RMR between the two methods (1569 vs. 1674 kcal/24 h, *p* = 0.12) was also observed. Different equations are often used for estimation of RMR, like the commonly used Harris-Benedict equation, sometimes with large errors [25]. Indirect calorimetry measures oxygen consumption and carbon dioxide production in a relaxed state. It is one of the most accurate methods for measurement of RMR and considered the “gold standard” [25, 26]. Otterstetter et al. [27] points out that Bod Pod underestimates RMR in comparison with indirect calorimetry, which is in accordance with our results.

Assessment of body composition in patients with obesity is difficult. Results from bioelectric impedance (BIA) must be interpreted with caution because the extracellular fluid volume is relatively greater in patients with obesity [28]. Computerized tomography, CT, is not possible in all body sizes and has the drawback of radiation exposure. Magnetic resonance imaging (MRI) is also limited to a certain body size, is associated with high costs, and is time-consuming. Hydrostatic weighing is considered “gold standard,” but it is time-consuming, often disliked by patients, and impractical in most patients with obesity [29, 30]. Dual-energy x-ray absorptiometry (DXA) is commonly used among researchers since it is very precise. However, DXA is limited by the size of the scanning area and by the whole body thickness in patients with obesity [31], and it also exposes the patients to radiation, although minor. In this study, we used air-displacement plethysmography, Bod Pod, since it is a fast, non-invasive, and reliable method to assess body composition in patients with obesity compared to the reference methods mentioned above [10, 29, 30, 32–34].

In our local and in the national database, BMI 5 years after RYGB and BPD/DS is on average 31 and 32 kg/m^2^, respectively [5]; for this reason, BMI 28–35 kg/m^2^ (31 ± 3 kg/m^2^ and 32 ± 3 kg/m^2^) was considered a representative postoperative BMI interval for this study. After the initial massive weight loss during the first 2 years, most patients reach a stable postoperative weight. The studied patients were weight stable, and after excluding six patients with weight regain after the last checkup, all studied individuals had a BMI below 35, the indication for bariatric surgery. Concerning the age interval (30–55 years), FFM is maintained up to 60 years of age [35], and all patients in this study were between 30 and 55 years old.

Some factors in the study need to be considered. Firstly, the groups were rather small. Our aim was, however, to examine whether RYGB and BPD/DS result in a non-desired body composition with regards to FFM%, by comparing these two groups to a non-operated control group with the same BMI. Secondly, in line with clinical experience, there were more men in the BPD/DS group (42% vs. approximately 20% in controls and RYGB), which could affect the results. However, we could not find a difference in FFM or FFM% comparing men and women separately. Thirdly, since the majority of weight loss is loss of FM, a previous higher BMI seen in the two bariatric groups might result in higher remaining FFM% after weight loss. The optimal control group would be individuals who had reached BMI 28–35 kg/m^2^ without surgery, with a previous BMI similar to that of the bariatric group’s preoperative BMI. However, such control group is very hard to find. Fourthly, detailed data of the patients’ lifestyle was not available, making it impossible to analyze any differences in exercise patterns between groups. The strength of the current study is the comparison of body composition and RMR in weight-stable RYGB and BPD/DS patients with a representative postoperative BMI to a non-operated control group with the same BMI of 28–35 kg/m^2^.

## Conclusion

Weight-stable patients with BMI 28–35 kg/m^2^ after RYGB and BPD/DS have a body composition and RMR similar to that of non-operated individuals within the same BMI interval.

## References

[1] Sjostrom L, Lindroos AK, Peltonen M, Torgerson J, Bouchard C, Carlsson B (2004). Lifestyle, diabetes, and cardiovascular risk factors 10 years after bariatric surgery. N Engl J Med.

[2] Sjostrom L, Peltonen M, Jacobson P, Ahlin S, Andersson-Assarsson J, Anveden A (2014). Association of bariatric surgery with long-term remission of type 2 diabetes and with microvascular and macrovascular complications. JAMA.

[3] Batsis JA, Lopez-Jimenez F, Collazo-Clavell ML, Clark MM, Somers VK, Sarr MG (2009). Quality of life after bariatric surgery: a population-based cohort study. Am J Med.

[4] Colquitt J, Clegg A, Loveman E, Royle P, Sidhu MK (2005). Surgery for morbid obesity. The Cochrane database of systematic reviews.

[5] Scandinavian Obesity Surgery Registry. SOReg, Arsrapport 2014 del 1–2. Available from: http://www.ucr.uu.se/soreg. Accessed 8 Mar 2016

[6] Marks BL, Rippe JM (1996). The importance of fat free mass maintenance in weight loss programmes. Sports Med.

[7] da Rocha EE, Alves VG, da Fonseca RB (2006). Indirect calorimetry: methodology, instruments and clinical application. Curr Opin Clin Nutr Metab Care..

[8] Chaston TB, Dixon JB, O'Brien PE (2007). Changes in fat-free mass during significant weight loss: a systematic review. Int J Obes.

[9] Bjermo H, Iggman D, Kullberg J, Dahlman I, Johansson L, Persson L (2012). Effects of n-6 PUFAs compared with SFAs on liver fat, lipoproteins, and inflammation in abdominal obesity: a randomized controlled trial. Am J Clin Nutr.

[10] Fields DA, Goran MI, McCrory MA (2002). Body-composition assessment via air-displacement plethysmography in adults and children: a review. Am J Clin Nutr.

[11] Tacchino RM, Mancini A, Perrelli M, Bianchi A, Giampietro A, Milardi D (2003). Body composition and energy expenditure: relationship and changes in obese subjects before and after biliopancreatic diversion. Metabolism.

[12] Benedetti G, Mingrone G, Marcoccia S, Benedetti M, Giancaterini A, Greco AV (2000). Body composition and energy expenditure after weight loss following bariatric surgery. J Am Coll Nutr.

[13] Strain GW, Gagner M, Inabnet WB, Dakin G, Pomp A (2007). Comparison of effects of gastric bypass and biliopancreatic diversion with duodenal switch on weight loss and body composition 1–2 years after surgery. Surg Obes Relat Dis.

[14] Carrasco F, Papapietro K, Csendes A, Salazar G, Echenique C, Lisboa C (2007). Changes in resting energy expenditure and body composition after weight loss following Roux-en-Y gastric bypass. Obes Surg.

[15] Das SK, Roberts SB, McCrory MA, Hsu LK, Shikora SA, Kehayias JJ (2003). Long-term changes in energy expenditure and body composition after massive weight loss induced by gastric bypass surgery. Am J Clin Nutr.

[16] Carey DG, Pliego GJ, Raymond RL (2006). Body composition and metabolic changes following bariatric surgery: effects on fat mass, lean mass and basal metabolic rate: six months to one-year follow-up. Obes Surg.

[17] Knuth ND, Johannsen DL, Tamboli RA, Marks-Shulman PA, Huizenga R, Chen KY (2014). Metabolic adaptation following massive weight loss is related to the degree of energy imbalance and changes in circulating leptin. Obesity.

[18] Heymsfield SB, Gonzalez MC, Shen W, Redman L, Thomas D (2014). Weight loss composition is one-fourth fat-free mass: a critical review and critique of this widely cited rule. Obes Rev.

[19] Kulovitz MG, Kolkmeyer D, Conn CA, Cohen DA, Ferraro RT (2014). Medical weight loss versus bariatric surgery: does method affect body composition and weight maintenance after 15% reduction in body weight?. Nutrition.

[20] Wadstrom C, Backman L, Forsberg AM, Nilsson E, Hultman E, Reizenstein P (2000). Body composition and muscle constituents during weight loss: studies in obese patients following gastroplasty. Obes Surg.

[21] Dixon JB, Lambert EA, Grima M, Rice T, Lambert GW, Straznicky NE (2015). Fat-free mass loss generated with weight loss in overweight and obese adults: what may we expect?. Diabetes Obes Metab.

[22] Vogels N, Diepvens K, Westerterp-Plantenga MS (2005). Predictors of long-term weight maintenance. Obes Res.

[23] Schmidt JB, Pedersen SD, Gregersen NT, Vestergaard L, Nielsen MS, Ritz C (2016). Effects of RYGB on energy expenditure, appetite and glycaemic control: a randomized controlled clinical trial. Int J Obes.

[24] Faria SL, Faria OP, Cardeal Mde A, de Gouvea HR, Buffington C (2012). Diet-induced thermogenesis and respiratory quotient after Roux-en-Y gastric bypass. Surg Obes Relat Dis.

[25] Boullata J, Williams J, Cottrell F, Hudson L, Compher C (2007). Accurate determination of energy needs in hospitalized patients. J Am Diet Assoc.

[26] Graf S, Karsegard VL, Viatte V, Heidegger CP, Fleury Y, Pichard C (2015). Evaluation of three indirect calorimetry devices in mechanically ventilated patients: which device compares best with the Deltatrac II((R))? A prospective observational study. Clin Nutr.

[27] Otterstetter R, Miller B, Fridline M, Boltz M, Faciana C, Scanlon K (2016). RMR estimation model accuracy using air displacement plethysmography-derived body composition measures in young adults. J Am Coll Nutr.

[28] Bioelectrical impedance analysis in body composition measurement: National Institutes of Health Technology Assessment Conference Statement. Am J Clin Nutr. 1996;64(3 Suppl):524S–32S. 10.1093/ajcn/64.3.524S 8780375

[29] Ginde SR, Geliebter A, Rubiano F, Silva AM, Wang J, Heshka S (2005). Air displacement plethysmography: validation in overweight and obese subjects. Obes Res.

[30] Petroni ML, Bertoli S, Maggioni M, Morini P, Battezzati A, Tagliaferri MA (2003). Feasibility of air plethysmography (BOD POD) in morbid obesity: a pilot study. Acta Diabetol.

[31] Laskey MA (1996). Dual-energy X-ray absorptiometry and body composition. Nutrition.

[32] Fields DA, Higgins PB, Radley D (2005). Air-displacement plethysmography: here to stay. Curr Opin Clin Nutr Metab Care.

[33] Tucker LA, Lecheminant JD, Bailey BW (2014). Test-retest reliability of the Bod Pod: the effect of multiple assessments. Percept Mot Skills.

[34] Lowry DW, Tomiyama AJ (2015). Air displacement plethysmography versus dual-energy x-ray absorptiometry in underweight, normal-weight, and overweight/obese individuals. PLoS One.

[35] Larsson I, Lissner L, Samuelson G, Fors H, Lantz H, Naslund I (2015). Body composition through adult life: Swedish reference data on body composition. Eur J Clin Nutr.

